# Optimization of oil extraction from giant bushel gourd seeds using response surface methodology

**DOI:** 10.1002/fsn3.341

**Published:** 2016-01-18

**Authors:** Yetunde Yemisi Popoola, Rahman Akinoso, Akeem Olayemi Raji

**Affiliations:** ^1^ Department of Food TechnologyUniversity of IbadanOyo StateNigeria; ^2^ Department of Food Agric and Biological EngineeringCollege of Engineering and TechnologyKwara State UniversityMalete, IlorinNigeria

**Keywords:** *Lagenaria siceraria*, oil expression, oil quality and optimization, oil yield

## Abstract

Gourd seeds have been identified as a source of edible oil, but there is sparse literature on the effect of processing factors on the characteristics of oil extracted from any *Lagenaria* spp. Optimization of oil extraction with the aid of expeller was achieved by applying response surface methodology. The variables were roasting temperature (87.70–172.0°C) and roasting duration (7.93–22.07 min), while the responses were oil yield and oil quality (free fatty acid, color, specific gravity, saponification value, moisture, and refractive index). Data obtained were analyzed at *P *< 0.05. Roasting conditions significantly influenced all the responses at *P *< 0.05. The optimum roasting condition was 100°C for 20 min, which gave 27.62% oil yield with good quality attributes (free fatty acid: 0.61%, color: 3.47 abs, specific gravity: 0.90 g/mL, saponification value: 289.66 mL, and refractive index: 1.47).

## Introduction

The demand for vegetable oils for food purposes requires a considerable expansion of oilseed crop production (CÇamas et al. 2007). Generally fat and oil are major sources of energy to human, generating greater amount of calories than carbohydrates in food (Dunford 2010). Aside from generating energy, they are known to be sources of some vitamins such as A, D, E, K, and essential fatty acids which the human body cannot synthesize. Fats and oil are derived from both animals and plants. The plant sources are more acceptable than the animal sources. This is due to the health issues, such as coronary heart diseases, associated with the animal sources of oil (Fellows 2000). Nutritionally, vegetable oil provides calories and vitamins in human diet in an easily digested form and at a lower cost (Dunford 2010). Despite the broad range of sources for vegetable oils, the world consumption is dominated by soybean, palm, rapeseed, and sunflower oils with 31.6, 30.5, 15.5, and 8.6 million tons consumed per year, respectively (Stevenson et al. 2007). In Nigeria, palm oil, groundnut oil, and coconut oil are the major oils consumed (Arinola and Ogunbusola 2013). These conventional sources of vegetable oil have little impact in meeting the increasing demand of vegetable oil for both human and industrial use. Hence, there is need to supplement the supplies with other sources, especially underutilized oilseeds.

Cucurbitaceae (gourds) are from an important and versatile family comprising hundreds of species of vine bearing, coil climbing tendrils and some of the most unusual fruits in the world (Ogundele and Oshodi 2010). Some important cucurbit family members include *Lagenaria siceraria* (calabash gourd), *Lagenaria sphaerica* (bottle gourd), *Citrullus colocynthis* (melon), *Cucumeropsis mannii* (white melon), *Cucurbita maxima* (pumpkin), *Cucurbita moschata* (musk melon), *Cucumis sativa* (*Ibo egusi*), and *Citrullus lanatus* (water melon). *Lagenaria siceraria* are tropical African plants grown in most parts of Nigeria for different purposes. In Yorubaland, gourds are used to store and serve palm wine, as musical instruments, as utensils, and even for traditional rites. However, some indigenous rural dwellers eat the seeds of these gourd plants as soup thickeners and are called *egusi igba*. *Lagenaria sphaerica* (African wine kettle) is otherwise called *Akeregbe* or *Agbe* in Yorubaland and *L. siceraria* (giant bushel gourd) is known as *Igbaje*. *Lagenaria siceraria* show amazing diversity, particularly with respect to fruit shape and size.

Pretreatments are conventional methods of preparing oilseeds for oil extraction. These include operations such as grinding, roasting, dehulling, flaking, cooking, or steaming (Akinoso and Raji 2011). They are carried out to fractionate oil intact bodies in order to enhance the release of oil during extraction (Kumar et al. 2009). The quality and quantity of the oil obtained from oilseeds by extraction processes are affected by various pretreatment conditions that seeds are subjected to prior to oil extraction. Most oilseeds and nuts are heat treated by roasting to liquefy the oil in the plant cells and facilitate its release during extraction (Cammerer and Kroh 2009).

Several studies have reported the chemical composition and oil characteristics of some *Lagenaria* species and other cucurbits from different regions by Akubugwo et al. (2009), Fokou et al.(2009), and Olaofe et al.(2012), but there is no existing literature on the effect of processing factors on the characteristics of oil extracted from any *Lagenaria* spp. This study was designed to investigate the effect of roasting temperature and duration on oil yield and quality of *L. siceraria* seeds.

## Materials and Methods

### Material preparation

Dried matured fruits of *L. siceraria* pods were purchased from “Bode” market in Ibadan, Oyo state, Nigeria. The fruits were broken to extract the seeds, the extracted seeds were sundried, sorted, and dehulled manually. The dehulled seeds were packaged in polyethene bags prior to further processing.

### Experimental design

A central composite rotatable design of response surface methodology was used as described by Montgomery (2005). Roasting temperature and duration were the variables, while the oil yield, specific gravity, color, free fatty acid (FFA), refractive index, moisture, and saponification value were the responses. Five levels of roasting temperatures and roasting durations were used and 13 samples were generated (Table 1). Roasting durations were 7.93, 10.0, 15.0, 20.0, and 22.07 min, while roasting temperatures were 87.7.0, 100.0, 130.0, 160.0, and 172.0°C.

**Table 1:** The experimental design and obtained values of the responses

Temperature (^°^C)	Duration (min)	Specific gravity (g/mL)	Moisture (%)	Yield (%)	Saponification value(mL)	Refractive index	Color (mg/L)	Free fatty acid (%)
87.70	20.00	0.93	0.15	29.50	323.87	1.4715	7.30	0.71
100.00	7.93	0.92	0.16	32.70	221.34	1.4715	6.20	2.82
100.00	10.00	0.84	0.13	30.00	271.79	1.4715	9.20	1.41
130.00	7.93	0.93	0.20	32.60	278.30	1.4715	4.10	2.82
130.00	15.00	0.93	0.22	22.60	275.05	1.4715	7.60	1.41
130.00	15.00	0.93	0.22	23.00	275.05	1.4715	7.70	1.41
130.00	15.00	0.93	0.22	20.90	262.20	1.4715	7.80	1.41
130.00	15.00	0.93	0.22	22.00	260.56	1.4715	8.00	1.41
130.00	15.00	0.93	0.22	23.50	263.67	1.4715	7.70	1.41
130.00	22.07	0.89	0.13	25.00	283.19	1.4715	8.00	0.71
160.00	10.00	0.88	0.15	31.30	322.25	1.4720	7.80	2.82
160.00	20.00	0.97	0.14	18.00	302.72	1.4720	10.80	0.71
172.00	15.00	0.95	0.18	18.70	275.04	1.4715	11.80	1.41

### Determination of moisture content

The gourd seeds were manually cleaned and the moisture content of the seeds was determined using AOAC (2005) method.

### Roasting of seeds

At a preset temperature, thin layers of 1000 g of each sample were heated on a heat conductor tray in an oven at a present temperature. The samples were heated at specified temperatures and durations stated in Table 1. A stop watch was used to confirm the duration.

### Oil extraction

The expeller used was Piteba screw oil expeller. It is a Holland handmade tool designed by Edwin Blaak. It is a manually operated expeller with a capacity of 5 kg. The barrel was heated for about 10 min before loading 1000 g of the preheated seeds into the expeller. The screw moved the seed toward the press cage outlet when the handle was turned. As a result of accumulation of seeds toward the press cage outlet, the seeds were ground and exposed to a very high pressure. With the help of the continuous heat supplied, the oil was expelled near the press cage outlet and ran against the direction of flow of the seeds. The extraction process took about 15 min for each sample.

### Physical and chemical evaluation of gourd oil

The extracted gourd oil was analyzed for some important physical and chemical properties. Oil yield, FFA, refractive index, moisture content, saponification value, color, and specific gravity of the oil were determined using standard American Oil Chemists' Society methods (AOCS 1997).

### Modeling and optimization

The choice of variable levels was selected with respect to preliminary trials. The data obtained were subjected to ANOVA and regression model generated. The process was optimized using a commercial statistical package (Design Experts, Stat‐Ease, Inc., Minneapolis, MN). Optimum process parameters were achieved by maximizing oil yield and saponification values, minimizing moisture content, FFA, and specific gravity, while the color and refractive index were kept in range.

## Results and Discussion

### Moisture content

The moisture content obtained for untreated gourd seeds was 5.50 ± 0.30% wet basis. The moisture content of the seed was within the range of 2.90–6.20% wet basis as reported by Akinoso and Raji (2011) for obtaining high yield and good quality of oil from oilseeds.

### Oil yield

**Figure 1:**
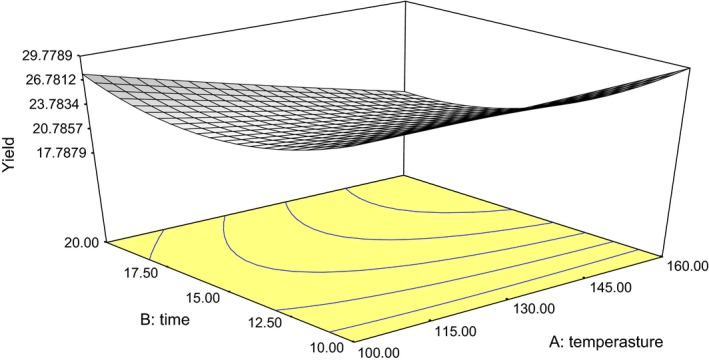
Plot of oil yield against roasting temperature and duration.

The oil yield ranged from 18.0% to 32.6% as presented in Table 1. The combined effect of roasting temperature and duration were significant on oil yield (*P *< 0.05). The coefficient of determination (*R* ^2^) was 0.96, which shows that the model fits well for the data. A response surface plot of the interaction is shown in Figure 1 and the quadratic model was the best to describe the relationship between the roasting conditions and the oil yield (eq. (1)). Lack‐of‐fit test for the model was not significant at *P *< 0.05 (Table 1). All the model terms were significant (*P *< 0.05). The range of oil yield (16.9–40%) obtained was found to be lower than the yields (38.10–43.65%) reported by Emmanuel et al.(2013) who worked on the properties of seed oils and variability in fatty acids composition of 10 *L. siceraria* cultivars. It was also lower than the values reported by Akinoso and Oni (2012) for melon seeds with a minimum oil yield of 44.0%. The average yield of the oil (25.4%) was found to be higher than yield recorded by Danjuma and Dandago (2009) and was within the range of the findings of Ajibola et al.(1990). The variation in oil yield might be attributed to differences in plant variety, cultivation, climate, ripening stage, harvesting time of seed, method and length of storage of seeds, and extraction method (Ottai et al. 2004).

(1) $$\begin{array}{cc} \text{OY}\;= & \;+42.575\;+\;0.133A\;-\;2.517B\;+\;2.1056E\;-\;004A^{2}\\ & \;+\;0.138B^{2}\;-\;0.018AB \end{array}$$

### Specific gravity

**Figure 2:**
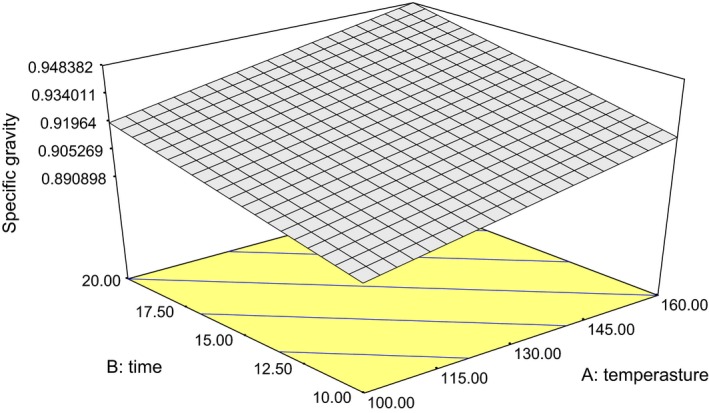
Plot of oil specific gravity against roasting temperature and duration.

The specific gravity of the oil samples varied from 0.841 to 0.971 g/mL. The treatment was found to be effectively significant at 95% confidence level. Linear model was found to be the best for predicting the relationship between the roasting conditions and specific gravity of the oil. High coefficient of determination *R* ^2^ (0.62) indicated that the model might fit well for the data. A visual illustration of the relationship is shown in Figure 2 and its mathematical relationship is expressed in equation (2). Significant effect (*P *< 0.05) was observed on the oil obtained. A reduction in the specific gravity of the oil was observed as the roasting duration and temperature increased. Reduction in specific gravity of the oil might be traced to thermal decomposition of fatty acid bond (Akinoso and Raji 2011). The specific gravity of the oil samples (0.841–0.971 g/mL) was within the range of the published data for three varieties of *Lagenaria* spp.: calabash (0.90 g/mL), bottle gourd (0.94 g/mL), and lump‐in‐neck (0.93 g/mL), respectively (Olaofe et al. 2012). It was also in close congruence with values reported by Emmanuel et al. (2013).

(2) SG=+0.814+4.791E−004A+2.874E−003B

### Color

**Figure 3:**
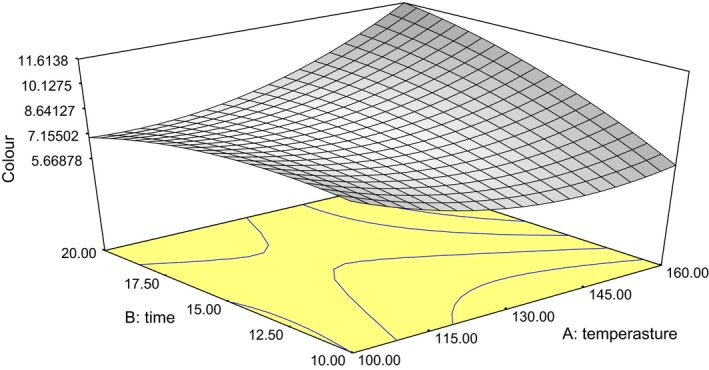
Plot of oil color against roasting time and roasting temperature.

The color of the oil ranged from 3.3 to 10.5 mg/L (Table 1). The combined effect of the roasting treatment was significant (*P *< 0.05) on color. The quadratic model was the best to predict the effect of the treatment on the color of the oil samples (eq. (3)). High coefficient of determination *R* ^2^ (0.92) indicated that the model had a good fit. The color intensity of the oil increased with increase in roasting temperature and roasting duration (Fig. 3). This was similar to the finding of Akinoso and Raji (2011) on effect of roasting conditions, on the color of locust seed oil. The increase in absorbance with an increase in roasting temperature and duration could be attributed to color formation by both nonenzymatic browning reaction and phospholipids degradation during roasting process (Mohagir et al. 2009).

(3) $$\begin{array}{cc} \text{CO}\;=\; & +45.755\;-\;0.578A\;-\;0.410B\;+\;1.610E\;-\;003A^{2}\\ & -\;0.0313B^{2}\;+\;0.0118AB \end{array}$$

### Free fatty acid

**Figure 4:**
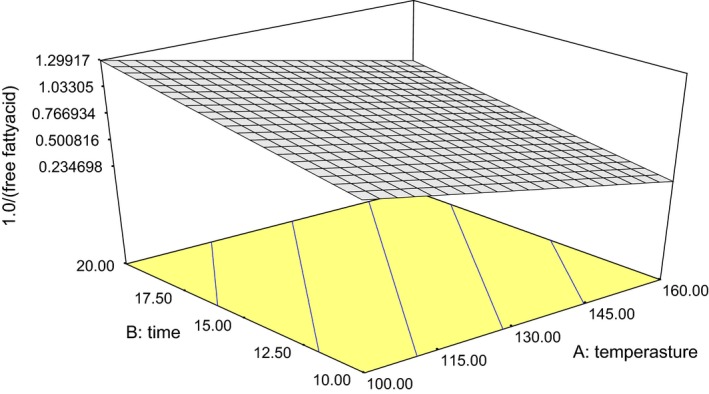
Plot of oil free fatty acid against roasting temperature and duration.

Free fatty acid of the samples ranged from 0.71% to 2.82% (Table 1). For best performance, there was a need to transform the model and the log of FFA was considered to establish the effect of roasting conditions on the FFA of the oil (eq. (4)). The model satisfied lack‐of‐fit test at *P *< 0.05 and the coefficient of determination *R* ^2^ was 0.51. The effect of the treatments was significant (*P *< 0.05) on the FFA of the oil samples of which an increase in roasting time and temperature led to an increase in FFA of the gourd oil samples (Fig. 4). The FFA obtained (0.71–2.82%) was found to be lower than 1.4–2.90% reported by Emmanuel et al.(2013) and 2.3–2.96% reported by Olaofe et al.(2012) for cultivars of *L. siceraria*. This might be as a result of varietal differences, storage conditions, and the extraction methods. Fokou et al.(2009) attributed high FFA of bottle gourd seed oils in their study to long storage period and method of processing of seeds. The low level of FFA of the samples suggested low level of hydrolytic and lipolytic activities (Akubugwo et al. 2009), thus the seed oil studied could be good source of industrial raw material.

(4) lnFFA=+3.164−0.02A−0.323B+2.319E−003

### Saponification value

**Figure 5:**
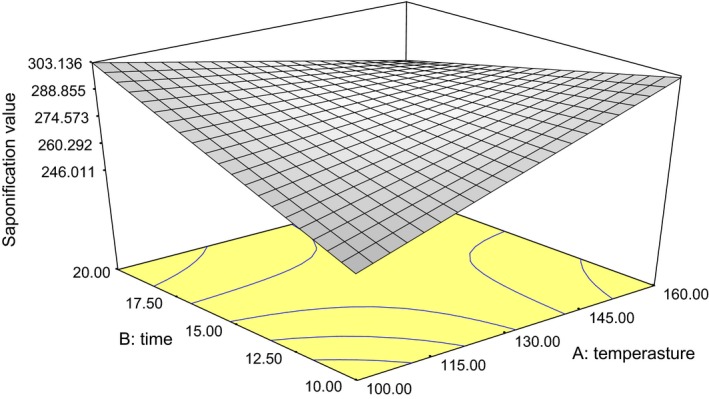
Plot of oil saponification value against roasting temperature and duration.

The saponification value for *Igba* varied from 221.34 to 323.87 mL (Table 1). There was a significant effect (*P* < 0.05) of roasting conditions on the saponification values of oil obtained from *Igba* seeds. A 2FI model was found to be applicable to express the relationship (Fig. 5), and the coefficient of determination *R* ^2^ is 0.56. Equation (5) shows the mathematical relationship between the saponification value of the oil and the roasting conditions. The average saponification value observed was greater than those reported for 10 cultivars of *L. siceraria* by Emmanuel et al. (2013) and a bit higher than standard value for vegetable oil which varied from 170 to 260 mg/g (Obasi et al. 2012).

(5) SV=−52.829+2.417A+20.517B−0.148AB

### Refractive index

**Figure 6:**
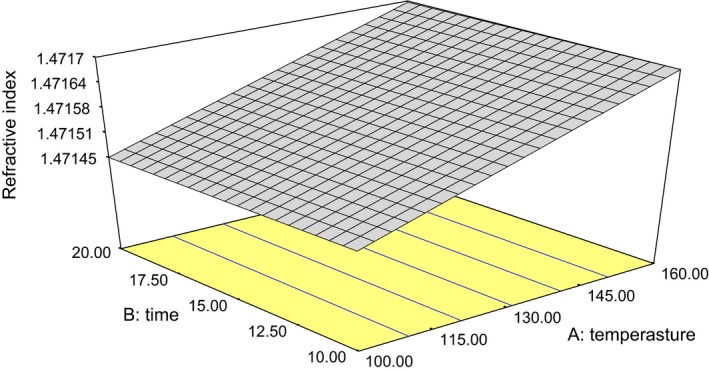
Plot of oil refractive index value against roasting temperature and duration.

The refractive index of oil samples ranged from 1.4715 to 1.4720, respectively (Table 1). The combined effect of the treatment was insignificant (*P* > 0.05) for refractive index of the oil samples. The linear model was best to predict the effect of the treatment of both oil samples (eq. (6)), and had *R* ^2^ of 0.30. Visual representations of the response surface plot of the interactions are shown in Figure 6. The refractive index of oils were found to be lower than the published data reported by Olaofe et al. (2012) for the oil obtained from *L. siceraria* seeds, extracted from different gourd shapes of calabash (1.49), bottle gourd (1.51), and lump‐in‐neck (1.49). It was also lower than the value (1.482) reported by Danjuma and Dandago (2009) for the extraction and characterization of calabash seeds oil, but higher than the values reported by Emmanuel et al.(2013) for 10 cultivars of *L. siceraria* which ranged from 1.34 to 1.45. The refractive index as a quality factor allows rapid sorting of oils suspected of adulteration and it is a measure of oil purity (Olaofe et al. 2012). The average value of refractive index (1.472) obtained for gourd seed oils in this study was indicative of the high degree of purity of the oil.

(6) RI=1.472+3.584E−006A−2.920E−005B

## Conclusions

The results obtained in this study showed that gourd seed is a potential source of vegetable oil. Roasting duration and temperature combinations influenced both oil yield and quality significantly at 95% confidence level. Models developed showed that gourd oil yield and oil quality (FFA, color, specific gravity, saponification value, moisture, and refractive index) were influenced by roasting temperature and duration. Six possible optimum solutions were found with desirability ranging from 0.65 to 0.67. The best of the six conditions was roasting at 100°C for 20 min, which gave optimum oil yield (27.62%) and good quality attributes (FFA: 0.61%, color: 3.47 abs, specific gravity: 0.90, saponification value: 289.66 mL, and refractive index: 1.47).

## Conflict of Interest

None declared.

## References

[fsn3341-bib-0001] Ajibola, O. O. , S. E. Eniyemo , O. O. Fasina , and K. A. Adeeko . 1990 Mechanical expression of oil from melon seeds. J. Agric. Eng. Res. 45:45–53.

[fsn3341-bib-0002] Akinoso, R. , and P. O. Oni . 2012 Optimization of solvent extracted melon seed using RSM. Eur. J. Lipid Sci. Technol. 114:607–611.

[fsn3341-bib-0003] Akinoso, R. , and A. O. Raji . 2011 Optimization of oil extraction from locust bean (*Parkia biglobosa*) using response methodology. Eur. J. Lipid Sci. Technol. 113:245–252.

[fsn3341-bib-0004] Akubugwo, C. G. , E. I. Chinenye , and A. E. Ugbogu . 2009 Nutritive value of *Lagenaria sphaerica* seed (wild bottle gourds) from South‐Eastern Nigeria. Pak. J. Nutr. 8:284–287.

[fsn3341-bib-0005] AOAC . 2005 International official methods of analysis, 18th ed. Association of Official Analytical Chemists, Gaithersburg, MD, USA.

[fsn3341-bib-0006] AOCS . 1997 Official methods and recommended practices of the American Oil Chemists Society. American Oil Chemist Society, Champaign.

[fsn3341-bib-0007] Arinola, S. O. , and E. M. Ogunbusola . 2013 Physicochemical characteristics and the effect of packaging materials on the storage stability of selected cucurbits oils. Am. J. Food Nutr. 1:34–37.

[fsn3341-bib-0008] CÇamas, N. , C. Irak , E. Esendal . 2007 Seed yield, oil content and fatty acids composition of safflower (*Carthamus tinctorius* L.) grown in Northern Turkey conditions. J. Fac. Agric. Univ. Ondokuz Mayis. 22:98–104.

[fsn3341-bib-0009] Cammerer, B. , and L. W. Kroh . 2009 Shelf life of linseeds and peanuts in relation to roasting. Food Sci. Technol. 42:545–549.

[fsn3341-bib-0010] Danjuma, M. N. , and M. A. Dandago . 2009 Extraction and characterization of calabash (*Lagenaria siceratia*) seed oil. Techno‐Science Africana J. 3:67–69.

[fsn3341-bib-0011] Dunford, N. 2010 “Oil and oilseed processing II” Food Technology Fact sheet; Robert M. Kerr Food and Agricultural Product Center (FAPC); 159.

[fsn3341-bib-0012] Emmanuel, E. E. , S. A. Bassey , and S. P. Nimmong‐uwem . 2013 *Lagenaria siceraria* (Mol.) standley – properties of seed oils and variability in fatty acids composition of ten cultivars. Int. J. Nat. Prod. Res. 3:102–106.

[fsn3341-bib-0014] Fellows, P. 2000 Food processing technology principles and practice. Woodhead Publishing Ltd, Cambridge, UK.

[fsn3341-bib-0015] Fokou, E. , M. B. Achu , G. Kansci , R. Ponka , M. Fotso , C. Tchiegang , et al. 2009 Chemical properties of some Cucurbitaceae oils from Cameroon. Pak. J. Nutr. 8:1325–1334.

[fsn3341-bib-0016] Kumar, S. , S. Debnath , and U. H. Hebbar . 2009 Pulsed infrared roasting of groundnuts and its quality. Int. J. Food Eng. 5:1–15.

[fsn3341-bib-0017] Mohagir, A. M. , R. Kamga , C. Kapseu , and C. F. Abi . 2009 Optimization of some pre‐treatments involved in the press extraction of Shea (*Vitellaria paradoxa Gaertner* F.) butter. Asian J. Appl. Sci. 2:372–384.

[fsn3341-bib-0018] Montgomery, D. C. 2005 Design and analysis of experiments: response surface method and designs. John Wiley and Sons Inc, New Jersey, USA.

[fsn3341-bib-0019] Obasi, N. A. , J. Ukadilonu , E. Eze , I. Akubugwo , and U. C. Okorie . 2012 Proximate composition, extraction, characterization and comparative assessment of coconut (*Cocos nucifera*) and melon (*Colocynthis citrullus*) seeds and seed oils. Pak. J. Biol. Sci. 15:1–9. 22530436 10.3923/pjbs.2012.1.9

[fsn3341-bib-0020] Ogundele, J. O. , and A. A. Oshodi . 2010 Proximate composition and some functional properties of three varieties of *Lagenaria Siceraria* . Research J. Agric. Biol. Sci. 6:108–112.

[fsn3341-bib-0021] Olaofe, O. , H. N. Ogungbenle , B. E. Akhadelor , A. O. Idris , O. V. Omojola , O. T. Omotehinse , et al. 2012 Physicochemical and fatty acids composition of oils from some legume seeds. Int. J. Biol., Pharm. Allied Sci. 1:355–363.

[fsn3341-bib-0022] Ottai, M. , A. Abdel‐Moniem , and R. A. El‐Mer‐gawi . 2004 Effect of variety and location on growth and yield components of Roselle, *Hibiscus sabdariffa* L. and its infestation with the spiny bollworm *Earias insulana* (Boisduval*)* . Arch. Phytopathol. Plant Prot. 37:215–231.

[fsn3341-bib-0023] Stevenson, D. G. , F. J. Eller , L. Wang , J. L. Jane , T. Wang , and G. E. Inglett . 2007 Oil and tocopherol content and composition of pumpkin seed oil in 12 cultivars. J. Agric. Food Chem. 55:4005–4013. 17439238 10.1021/jf0706979

